# Randomized Clinical Trial to Compare Cryopreserved and Lyopreserved Umbilical Cord Tissue to Treat Complex Diabetic Foot Wounds

**DOI:** 10.1177/15347346241273282

**Published:** 2024-12-05

**Authors:** Lawrence A. Lavery, Mehmet A. Suludere, Matthew J. Johnson, Amanda L. Killeen, Katherine M. Raspovic, Peter A. Crisologo, Arthur N. Tarricone

**Affiliations:** 1 Department of Plastic Surgery, 12334 University of Texas Southwestern Medical Center, Dallas, Texas, USA; 2 Department of Orthopedic Surgery, 12334 University of Texas Southwestern Medical Center, Dallas, Texas, USA; 3 College of Podiatric Medicine, 441554 University of Texas Rio Grande Valley School of Medicine, Harlingen, Texas, USA; 4 Department of Orthopedic Surgery, University of Texas Health Science Center, San Antonio, Texas, USA

**Keywords:** diabetic foot ulcers, lower extremity wound, umbilical cord tissue, infection, wound

## Abstract

To compare the incidence of infection, wound closure and time to wound closure in patients treated with cryopreserved (CPUT) and lyopreserved umbilical tissue (LPUT) in complex diabetic surgical wounds. This single-blinded 12-week randomized clinical trial compared cryopreserved and lyopreserved amniotic cord tissue to treat complex diabetic foot wounds. LPUT or CRAT was applied at baseline and again after four weeks. We enrolled subjects with UT2A-D and 3A-D wounds (depth to tendon, muscle, or bone with infection and/or PAD) and excluded subjects with ABI < 0.5 or TBI < 0.3, untreated osteomyelitis, and autoimmune diseases. We used a 3-D camera to evaluate wound area and volume. The mean baseline wound areas were 12.9 ± 10.7 cm^2^ for CPUT and 11.7 ± 7.0 cm^2^ for LPUT. The mean baseline wound volume was 7.5 ± 8.1 for CPUT and 9.2 ± 10.2 cm^3^ for LPUT. There was no difference between CPUT and LPUT in wound closure (36.8% vs 19.0%, *P* = .21) or infection (10.5% vs 4.8%, *P* = .60). There was no difference in mean wound area reduction between CPUT and LPUT (75.9 ± 32.3% vs 65.5 ± 38.4%, *P* = .41), nor in mean volume reduction (85.0 ± 30.8% vs 79.9 ± 31.9%, *P* = .61). In addition, there was no difference in wound closure trajectories for changes in area (*P* = .75) or volume (*P* = .43). Cryopreserved and lyopreserved amniotic tissue provided similar results in patients with complex diabetic foot wounds.

## Introduction

Amniotic tissue products have been available for more than a decade. This technology has been successfully used in randomized clinical trials for patients with full thickness diabetic foot ulcers (UT1A and UT1C ulcers)^1^ and in prospective cohort studies in patients with diabetes and complex surgical wounds with exposed tendon, muscle and bone.^2^ Initially, amniotic tissue products were thin and designed for weekly clinic applications with a focus on treating full thickness ulcers. In order to maintain viable endogenous growth factors and stem cells some of the first applications of amniotic tissue were cryopreserved. This technology required that the product was transported and stored in a −80 °C freezer and thawed immediately before it was used in the operating room.

A novel lyopreservation technology was used to maintain cell viability.^3^ In previous work, lyopreserved and cryopreserved amniotic tissue technology has been shown to have similar endogenous cell viability and clinical results. In a 12-week randomized clinical trial, we compared cryo- and lyopreserved amniotic tissue (Grafix, Smith + Nephew, Fort Worth, Texas) in UT1A and UT1C ulcers in which subjects received weekly applications (Grafix, Smith + Nephew, Fort Worth, Texas). There were no differences in the incidence of closure and time to closure. In addition, the lyopreserved technology had sustained living cells and tissues equivalent to cryopreserved amniotic tissue.^4,5^ To treat wounds with exposed deep structures, a cryopreserved product from amniotic umbilical cord (Stravix, Smith + Nephew, Fort Worth, Texas) was introduced. This construct is strong enough to be sutured into the wound bed, cover deep structures, and stay in place for several weeks. A lyopreserved version of this technology was subsequently developed to make storage and access easier. We hypothesized that robust umbilical cord tissue technology could be used in complex diabetic foot wounds, and that the new lyopreserved technology would be as effective as cryopreserved products. The goal of this study was to compare cryopreserved umbilical cord tissue (CPUT) and lyopreserved umbilical cord tissue (LPUT) in complex diabetic surgical wounds with exposed muscle, tendon, or bone. We evaluated the incidence of wound closure, time to closure, infection, wound area reduction (WAR) and wound volume reduction (WVR).

## Methods

### Trial Design

This was a patient-blinded randomized clinical trial that was approved by the local Institutional Review and reported on clinicaltrials.gov (NCT04405765). We enrolled 46 patients from two Hospital Centers.

### Population

We included patients over the age of 21with diabetes mellitus as defined by the American Diabetes Association^6^ with post-operative wounds >4 cm^2^, ankle-brachial index ≥ 0.5 or toe pressure >30 mm Hg, and wounds indicated for treatment with negative pressure wound therapy. We used the University of Texas Wound Classification to describe the severity of wounds,^7^ corresponding to UT 2-3 A-D. These included infected wounds that extended to tendon, fascia muscle or bone. We excluded patients with active Charcot neuroarthropathy, untreated bone of soft tissue infection, pregnant or nursing mothers, developmental disability or psychological disorder that would prevent informed consent, and alcohol or substance abuse that could impair consent or participation.

We enrolled patients hospitalized for moderate and severe diabetic foot infections.^8^ We used the foot infection classification developed by the International Working Group on the Diabetic Foot to classify the presence and severity of infection during the follow-up.^8^

Sensory neuropathy was evaluated with a 10-g Semmes Weinstein monofilament and vibration perception threshold testing (VPT) (Biothesiometer, Xilas Medical Inc., San Antonio, Texas) at the great toe and medial malleolus. Sensory neuropathy was defined as either VPT >25 volts or any site missed with 10-g monofilament. Peripheral arterial disease (PAD) was defined by ankle brachial index <0.9 (ABI). The systolic pressure from dorsalis pedis and posterior tibial arteries was used to calculate ABI for each artery. We also evaluated systolic toe pressures, toe/brachial indices and waveforms for the dorsalis pedis and posterior tibial arteries. We recorded wound area and volume using a 3D measurement device (inSight, eKare, Fairfax, Virginia). This device has been shown to be reproducible and reliable.^9^ Images were taken by the research coordinators and wound edge tracings were approved or re-traced by the investigator prior to recording the area and volume.

Per our standard protocol, patients were initially taken to the operating room for incision and drainage to excise infected and non-viable tissue. Patients then returned to the operating room in 48-72 h for a second surgery. Once the infection was resolved, patients were randomized to receive either cryopreserved or lyopreserved amniotic tissue in the operating room on their last surgery. We used a computer-generated randomization list with randomization assignments sealed in opaque envelopes that were consecutively numbered. Once the subject met the study size criteria during the final evaluation in the operating room, the envelope was opened, and treatment was instituted based on treatment assignment. Patients were then seen in the clinic weekly for 12 weeks. Data collected during the study included demographics, co-morbidities, history of drug, alcohol, tobacco, wound location, and wound duration.

### Intervention

The tissue used in this study was from cryopreserved and lyopreserved umbilical cord tissue. In cryopreserved technology, umbilical cord tissue is minimally manipulated and stored at ultra-low temperatures. Lyophilization is a process of optimally removing the water content of tissue to dry the frozen specimen.^10^ The desiccated tissue can then be stored at room temperature for prolonged periods.

### Endpoints

Wounds were sharply debrided as deemed necessary by the treating physician and offloaded using a healing sandal (Med-Surg Post-Operative Shoe, Darco, Huntington, West Virginia), removable cast boot (DH Offloading Walker, Össur, Reykjavík, Iceland), or a total contact cast, based on the location of the ulcer and the postural stability or fall risk of the subject. We defined complete wound closure as 100% epithelialization with no drainage. In addition, we used 90% wound area reduction to differentiate responders and non-responders to compare wound healing trajectories.

### Statistical Design

Intent to treat analysis was used. Study variables were summarized as mean and standard deviations for continuous variables and proportional comparisons for categorical variables. All continuous variables were tested for normality using quantile-quantile, histogram, and Shapiro-Wilk analysis. As most of these variables did not follow normal distribution, descriptive statistical analyses were utilized to determine median values of continuous variables with the 25th to 75th interquartile range (IQR) and frequencies of categorical variables. Continuous variables between groups were compared using either the Student's *t*-test or Mann-Whitney *U*-test, and categorical variables were analyzed using Pearson χ^2^ test or Fisher exact test (SPSS, IBM, Chicago, Illinois). This was a pilot study, so we did not have a sample size justification. We used an alpha of *α *= 0.05.

## Results

**Figure 1.**
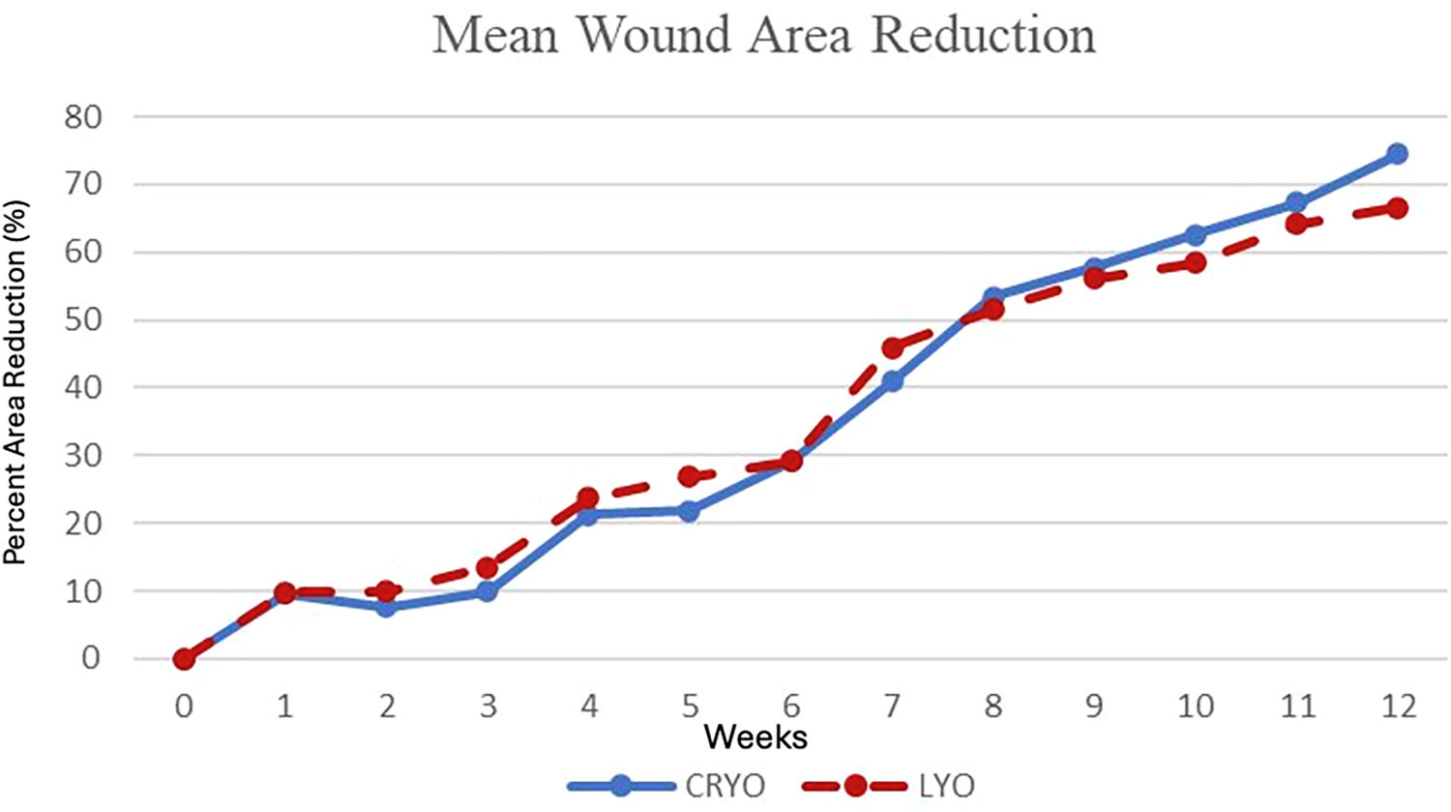
Weekly mean percent wound area reduction in patients treated with cryopreserved and lyopreserved amniotic tissue. There was no difference in trajectories between the treatment arms.

**Figure 2.**
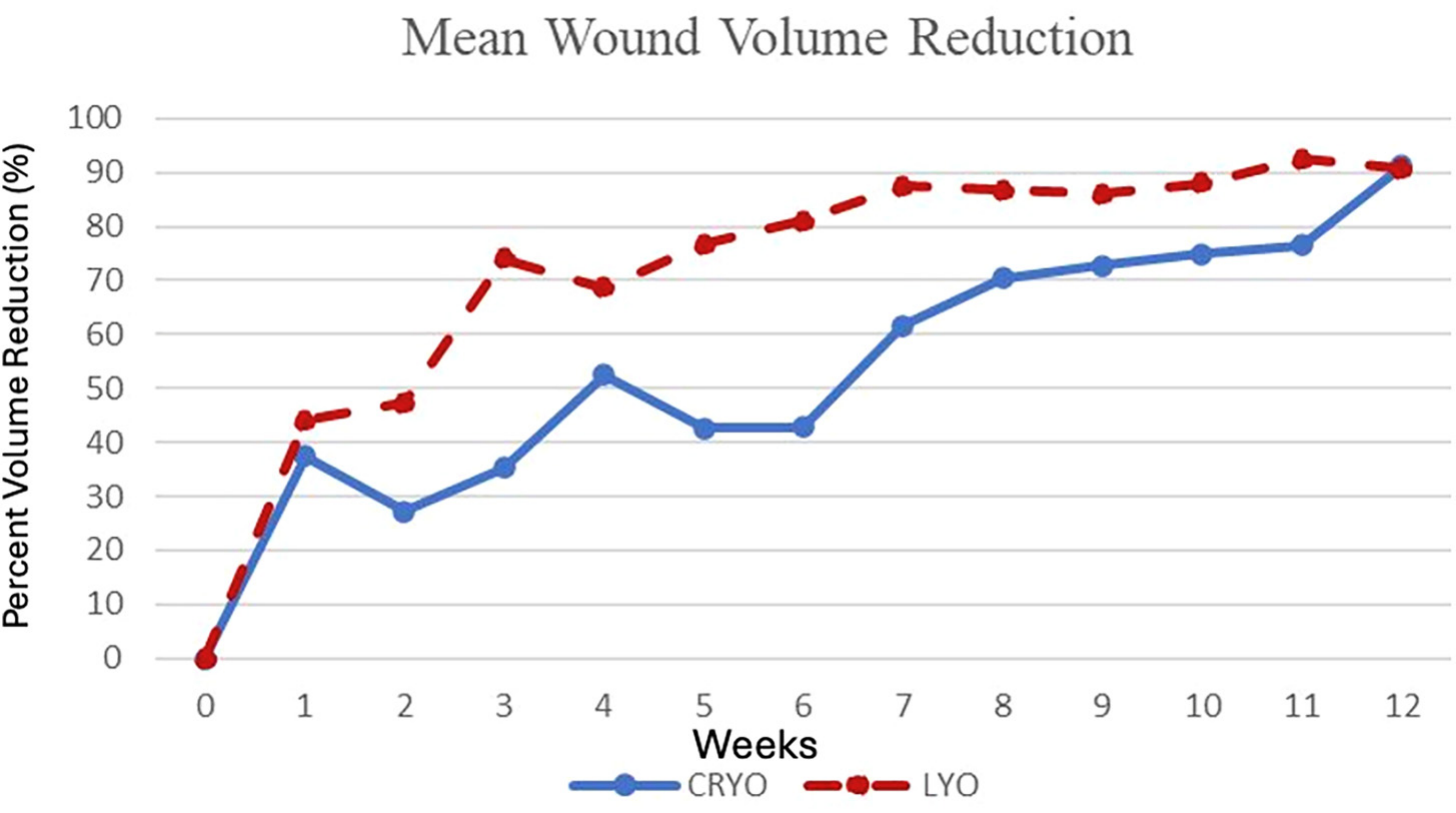
Weekly percent wound volume reduction in patients treated with cryopreserved and lyopreserved amniotic tissue. There was no difference in the trajectories between the treatment arms.

**Figure 3.**
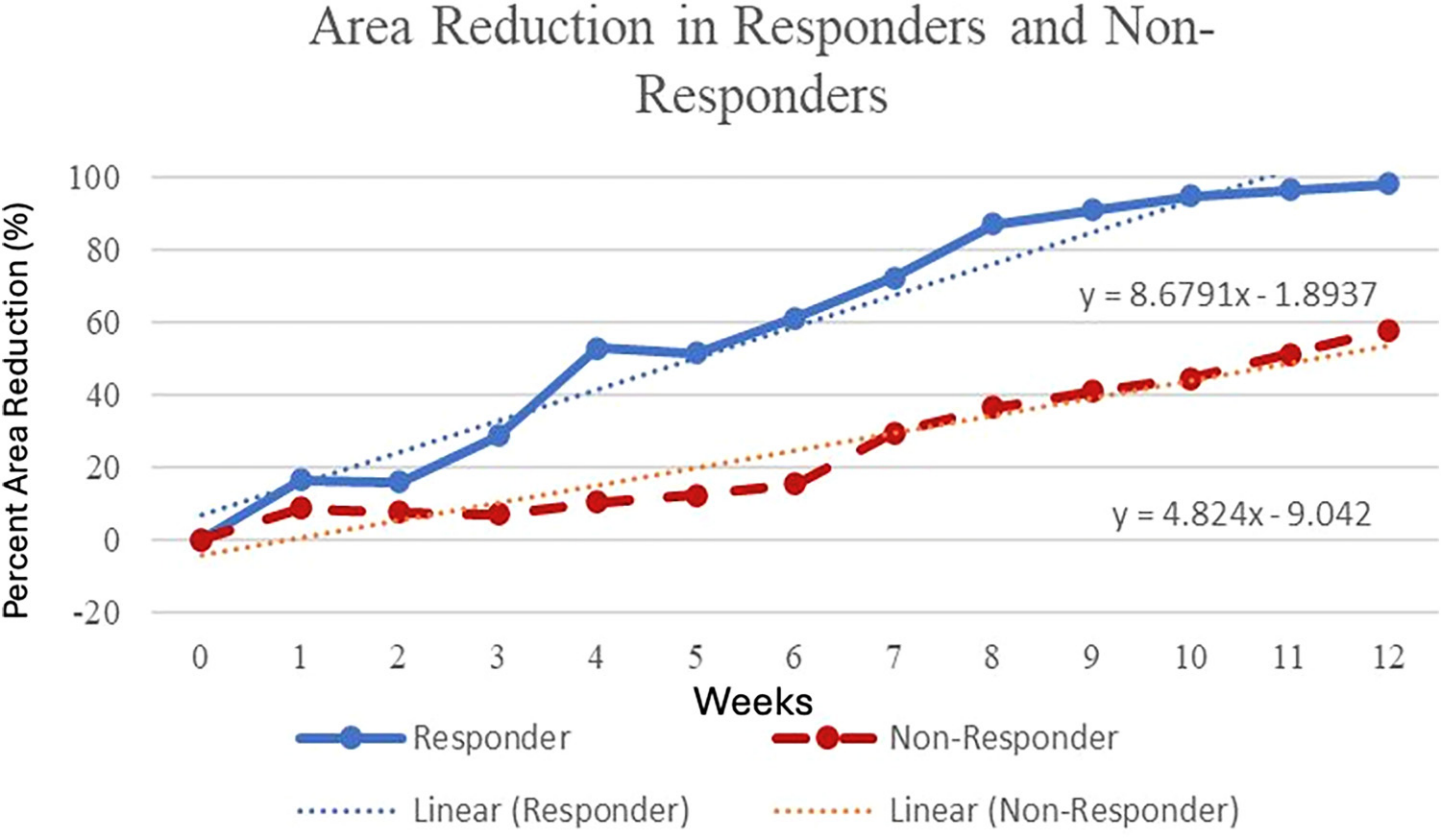
Weekly percent wound area reduction comparing responders and non-responders.

We enrolled 46 subjects in this study. Six patients failed screening and 40 were randomized (Figure 1). Patient characteristics are provided in Table 1. The mean baseline wound areas were 12.9 ± 10.7 cm^2^ for CPUT and 11.7 ± 7.0 cm^2^ for LPUT. The mean baseline wound volume was 7.5 ± 8.1 cm^3^ for CPUT and 9.2 ± 10.2 cm^3^ for LPUT. There was no difference between CPUT and LPUT in wound closure (36.8% vs 19.0%, *P* = .21) or infection of the study wound (10.5% vs 4.8%, *P* = .60). Three patients had infections of the study wound, and two additional infections were observed that did not involve the study wound. Four infections were mild and one was moderate, based on IWGDF infection classification. There were five infections in four patients. One patient had a moderate infection of the study wound in week 1 and a subsequent infection involving a separate ulcer later in the study. There was no difference in mean wound area reduction between CPUT and LPUT (75.9 ± 32.3% vs 65.5 ± 38.4%, *P* = .41, Figure 2), nor in mean volume reduction (85.0 ± 30.8% vs 79.9 ± 31.9%, *P* = .61, Figure 3). In addition, there was no difference in the mean weekly wound healing rate in area (1.1 ± 0.67 cm^2^ /week vs 0.75 ± 0.63 cm^2^/week, *P* = .08) or volume (0.70 ± 0.85 cm^3^ /week vs 0.92 ± 1.3 cm^3^/week, *P* = .21).

**Table 1.** Continuous Variables Listed as Mean ± SD. Area and Volume Listed as Median, Mean ± SD. Dichotomous Variables Listed as Number (Percent).

	Cryopreserved N = 19	Lyopreserved N = 21	*P* value
Demographics			
Male	16 (84.2)	17 (81.0)	.79
Age	55.4 ± 6.4	50.5 ± 11.5	.11
BMI (kg/m^2^)	30.9 ± 5.7	35.7 ± 7.1	.03
Race/Ethnicity			
Caucasian	1 (5.3)	5 (23.8)	.22
African American	5 (26.3)	6 (28.6)	.87
Hispanic	9 (47.4)	10 (47.7)	.99
Other	4 (21.1)	0 (0)	.10
Congestive Heart Failure	4 (21.1)	4 (19.0)	.87
Hypertension	17 (89.5)	15 (71.4)	.15
Chronic Kidney Disease	10 (52.6)	7 (33.3)	.22
Dialysis	0 (0.0)	1 (4.8%)	.53
Peripheral Artery Disease (ABI <0.9)	7 (36.8)	3 (14.3)	.21
Diabetes—type 2	18 (94.7)	21 (100)	.48
Diabetes Duration	12.5 ± 8.4	14.5 ± 12.2	.56
Diabetes Treatment			
Insulin	5 (26.3)	6 (28.6)	.87
Orals	6 (31.58)	1 (4.8)	.04
Combination therapy	7 (36.8)	13 (61.0)	.11
Diet	1 (5.3)	1 (4.8)	1.00
Tobacco user, current or previous	16 (84.2)	18 (85.7)	1.0
Alcohol user, current or previous	17 (89.5)	26 (76.2)	.23
History Foot Ulcer - same foot	4 (21.1)	1 (4.8)	.17
History Foot Ulcer - other foot	3 (15.8)	7 (33.3)	.28
History Foot Ulcer—both feet	2 (10.5)	5 (23.8)	.41
History of Osteomyelitis	9 (47.4)	11 (52.4)	.75
Wound Location			
Plantar foot	8 (42.1)	14 (66.7)	.12
Dorsal foot	5 (26.3)	2 (9.5)	.16
Medial foot	2 (10.5)	2 (9.5)	.92
Lateral foot	4 (21.1)	3 (14.3)	.57
Sensory Neuropathy	17 (89.5)	21 (100.0)	.25
10-gram Monofilament	16 (84.2)	18 (85.7)	.89
VPT Testing Great toe (volts)	49.6 ± 26.5	53.5 ± 29.8	.69
VPT Testing Ankle (volts)	39.5 ± 24.0	46.3 ± 26.1	.42
ABI Dorsalis Pedis	1.1 ± 0.3	1.1 ± 0.3	.93
ABI Posterior Tibial	1.0 ± 0.3	1.1 ± 3.7	.46
Toe Brachial Index	0.6 ± .02	0.7 ± 0.3	.35
Wound Area (cm^2^)	13.3, 12.9 ± 10.7	9.9, 11.7 ± 7.0	.68
Wound Volume (cm^3^)	3.6, 7.5 ± 8.1	5.2, 9.2 ± 10.2	.49
Infection—study wound	2 (10.5)	1 (4.8)	.60
Infection—non-study wound	1.0 (5.3)	1 (4.8)	1.0
IWGDF mild	2 (510.6)	2 (9.5)	1.0
IWGDF moderate	1 (5.3)	0 (0)	.48
Offloading			
Removable boot	15 (78.9)	11(52.4)	.08
Healing sandal	3(15.8)	3 (14.3)	.89
Total contact cast	0	3(14.3)	.23
Other	1(5.3)	4(19.0)	.34
Laboratory values at admission			
Glycated Hemoglobin	9.1 ± 2.0	9.9 ± 2.9	.36
White Blood Cell Count	12.0 ± 3.4	12.2 ± 4.4	.86
Erythrocyte Sedimentation Rate	79.5 ± 40.2	81.8 ± 37.9	.88
C-Reactive Protein	51.3 ± 90.7	31.93= ± 56.6	.48

**Figure 4.**
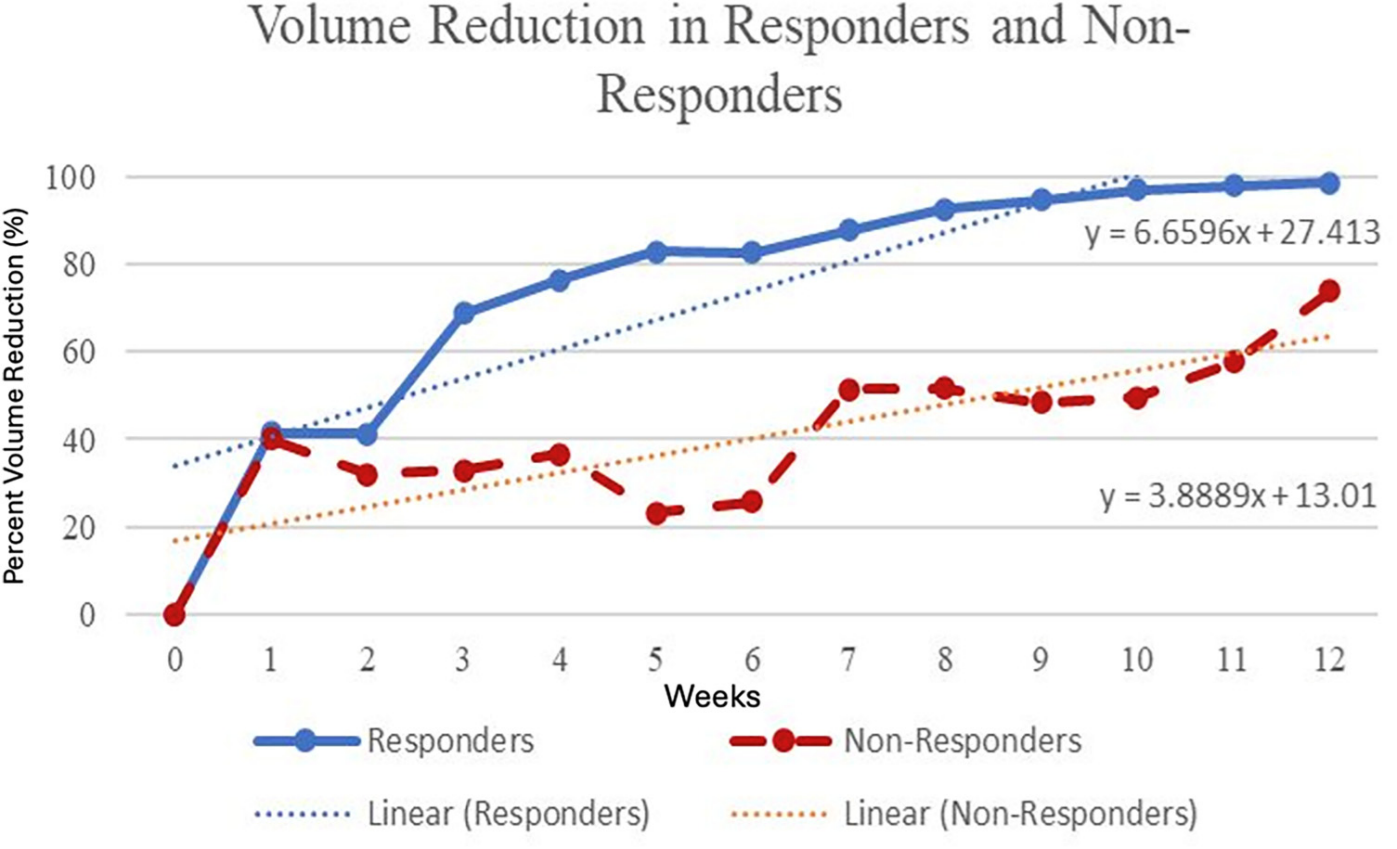
Weekly percent wound volume reduction comparing responders and non-responders.

**Figure 5.**
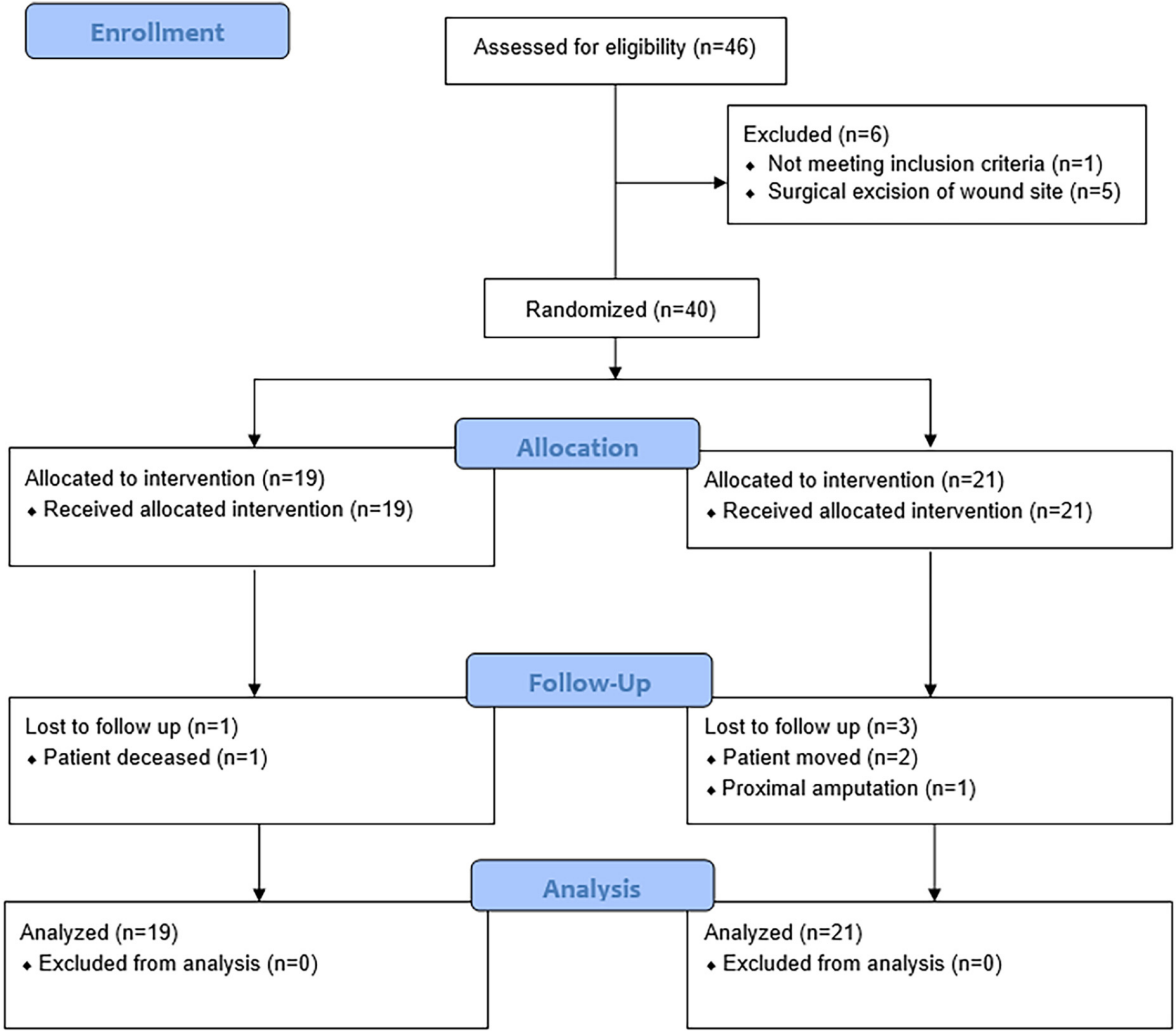
CONSORT flow chart.

We compared clinical and treatment variables in responders and non-responders based on a WAR <90% (Table 2). There were 23 (57.5%) patients who had a wound area reduction (WAR) of <90% at the end of the study. The only significant difference between the two groups was the baseline wound area (8.3 ± 4.8 vs 15.2 ± 10.1 cm^2^, *P* < .001). There was no difference in volume, mean weekly closure rate when comparing area (0.98 ± 1.0 cm^2^ /week vs 0.81 ± 0.74 cm^2^ /week, *P* = .30, Figure 4) or volume (0.74 ± 1.02 cm^3^/week vs 0.63 ± 1.6 cm^3^/week *P* = .40, Figure 5). All of the subjects who had infections were in the non-responder group, and none of these patients dropped out of the study.

**Table 2.** Continuous Variables Listed as Mean ± SD. Dichotomous Variables Listed as Number (Percent). BMI = body mass index, ABI = ankle brachial index, UT = umbilical cord tissue.

	Responders N = 17	Non-Responders N = 23	*P* value
Male	14 (73.7)	17 (89.5)	.58
Age	53.1 ± 8.8	51.5 ± 10.3	.60
BMI (kg/m^2^)	34.1 ± 6.9	32.9 ± 6.6	.59
Cryopreserved UT	8 (47.1)	11 (47.8)	1.0
Years with Diabetes	16.5 ± 8.0	11.4 ± 11.6	.14
Peripheral Arterial Disease	5 (29.4)	5 (21.7)	.58
Chronic Kidney Disease	8 (34.8)	8 (34.8)	.52
ABI—Posterior Tibial	1.1 ± 0.25	1.1 ± 0.25	.92
ABI—Dorsalis Pedis	1.1 ± 0.21	1.1 ± 0.28	.70
Toe Brachial Index	0.65 ± 0.21	0.63 ± 0.33	.83
Offload boot/cast	11 (64.7)	18 (78.3)	.48
Infection any site	0 (0)	5 (21.7)	.06
Infection—study wound	0 (0)	3 (13.0)	.25
Baseline wound area (cm^2^)	8.3 ± 4.8	15.2 ± 10.1 cm^2^	*P* < .001
Baseline wound volume (cm^3^)	7.04 ± 10.27	9.37 ± 8.53	.44

## Discussion

The results of this pilot study showed no differences in outcomes in patients that received cryopreserved and lyopreserved amniotic tissue products. The rate of wound closure, time to closure, and re-infections during the 12-week evaluation period were not significantly different in the treatment groups. In addition, the weekly wound area reduction and wound volume reduction show similar trajectories during the 12-week evaluation. The lyopreserved technology provides easier access to the product since it can be stored in the operating theatre without requiring a freezer.

Complex wounds with exposed tendon, muscle, and bone are especially challenging to treat. There are only a few RCTs that evaluate complex diabetic foot wounds with exposed tendon, muscle or bone.^11–15^ This study is novel in that we evaluated wound area and volume. Most studies that evaluate complex wounds only report changes in wound area. The mean wound area was large compared to RCTs that enrolled UT1A and UT1C DFUs (diabetic foot ulcer). In most of these studies the average wound size was 2-4 cm^2^.^16^ In contrast, the average wound area in this study was 12 cm^2^. Other RCTs that evaluate complex diabetic foot wounds include wounds that range from 10 to 21 cm^2^.^11–13^

In this study, the incidence of re-infection was very low during the 12-week evaluation period compared to other DFU RCTs and cohort studies of complex diabetic foot wounds. Infection is a common complication in people with diabetes and foot wounds, even when the wounds do not involve exposed deep structures. In the standard of care arm in DFU RCTs that include included UT1A and UT1C ulcers, 18%–36% of patients develop an infection.^1^ There were significantly fewer infections in the active treatment arms, and these subjects have a higher incidence and faster time to heal (18%–22%).^1,17,18^ The lower infection rate could be related to the shorter exposure period. Unfortunately, many amniotic studies do not report infections.^19^ The current study included UT2A-D and UT3A-D wounds. Despite having larger and more severe wounds with recent infection, there was a very low incidence of infection of the study wound in both groups (7.5%). In a retrospective cohort study of complex wounds with exposed tendon, muscle or bone, the rate of re-infection after the index hospitalization was 47.3% during the next year.^20^ In RCTs that evaluated^11–13,15^ complex diabetic foot wounds, the reported rate of re-infection ranged from 21.3 to 26.7% in 12 to 16 weeks.

While we do not have a direct comparison of standard care to evaluate infection in this study, the rate of infection is very low. This may be due to the umbilical cord tissue serving as a barrier against bacterial infiltration or the tissue itself secreting peptides like human beta-defensins that contribute an antimicrobial effect. Mao et al demonstrated secreted factors were responsible for multiple log reductions in *Pseudomonas, Staphylococcus,* and *Streptococcus*, the most common pathogens in diabetic foot infections.^21^ Their group also showed activity against *E faecium, K. pneumoniae, A baumanii,* and E*. aerogenes* is maintained, even when placental-derived tissue has been processed with dehydration or freezing.^22^

There are strengths and limitations to this study. The purpose of this study was to compare results in complex wounds treated with cryopreserved and lyopreserved technologies; therefore, there was no control arm that received standard of care. This might be seen as a limitation, but the standard of care arm did not serve the objective of this pilot study. This was a small proof of concept study with limited sample size. It could be argued that a larger sample size would have shown a difference in wound closure. When we calculated a sample size estimate using the outcomes observed in the study, we would have needed to enroll 776 subjects in each arm of the study with a power of 80% and an alpha of 0.05.

## Conclusion

In this pilot study there were no differences in clinical outcomes or complications in complex diabetic foot wounds treated with cryopreserved and lyopreserved tissue. There was a very low rate of infection (7.5%) and a very fast rate of wound area reduction (0.90 cm^2^ per week) and volume reduction (0.80 cm^3^/week).
